# Characterization of Bioactive Compounds from *Acacia concinna* and *Citrus limon*, Silver Nanoparticles’ Production by *A. concinna* Extract, and Their Biological Properties

**DOI:** 10.3390/molecules27092715

**Published:** 2022-04-22

**Authors:** Ibrahim Ahmed Shaikh, Uday M. Muddapur, Zabin K. Bagewadi, Sneha Chiniwal, Mohammed M. Ghoneim, Mater H. Mahnashi, Fahad Alsaikhan, Deepak Yaraguppi, Francois N. Niyonzima, Sunil S. More, Basheerahmed Abdulaziz Mannasaheb, Amer Al Ali, Abdulaziz Asiri, Aejaz Abdullatif Khan, S. M. Shakeel Iqubal

**Affiliations:** 1 Department of Pharmacology, College of Pharmacy, Najran University, Najran 66462, Saudi Arabia; i.ibrahimshaikh09@gmail.com; 2 Department of Biotechnology, KLE Technological University, BVB Campus, Hubballi 580031, Karnataka, India; zabinb@gmail.com (Z.K.B.); snehachiniwal@gmail.com (S.C.); deepak.yaraguppi@kletech.ac.in (D.Y.); 3 Department of Pharmacy Practice, College of Pharmacy, AlMaarefa University, Dariyah, Riyadh 13713, Saudi Arabia; mghoneim@mcst.edu.sa (M.M.G.); bmannasaheb@mcst.edu.sa (B.A.M.); 4 Pharmacognosy and Medicinal Plants Department, Faculty of Pharmacy (Boys), Al-Azhar University, Cairo 11884, Egypt; 5 Department of Pharmaceutical Chemistry, College of Pharmacy, Najran University, Najran 66462, Saudi Arabia; matermaha@gmail.com; 6 Department of Clinical Pharmacy, College of Pharmacy, Prince Sattam Bin Abdulaziz University, Alkharj 11942, Saudi Arabia; fsaikhan@hotmail.com; 7 Department of Math, Science and PE, CE, University of Rwanda, Rwamagana 55, Rwanda; niyofra@yahoo.com; 8 School of Basic and Applied Sciences, Dayananda Sagar University, Bangalore 560111, Karnataka, India; sunilmorey@gmail.com; 9 Department of Clinical Laboratory Sciences, Faculty of Applied Medical Sciences, University of Bisha, 255, Al Nakhil, Bisha 67714, Saudi Arabia; ameralali@ub.edu.sa; 10 Faculty of Applied Medical Sciences, University of Bisha, 255, Al Nakhil, Bisha 67714, Saudi Arabia; amfasiri@ub.edu.sa; 11 Department of General Science, Ibn Sina National College for Medical Studies, Jeddah 21418, Saudi Arabia; aeju_kh@yahoo.com (A.A.K.); shakeeliqubal@gmail.com (S.M.S.I.)

**Keywords:** medicinal plants, *Acacia concinna*, *Citrus limon*, phytochemical screening, antidiabetic, anticancer

## Abstract

The applications of bioactive compounds from medicinal plants as therapeutic drugs are largely increasing. The present study selected the bioactive compounds from *Acacia concinna* (*A. concinna*) and *Citrus limon* (*C. limon*) to assess their phytochemicals, proteins, and biological activity. The plant material was collected, and extraction performed as per the standard procedure. Qualitative analysis was undertaken, and identification of functional organic groups was performed by FTIR and HPLC. Antibacterial, anticancer, antioxidant, antihyperglycemic, antihyperlipidemic, and inhibition kinetics studies for enzymes were performed to assess the different biological activities. Flavonoids and phenols were present in a significant amount in both the selected plants. *A. concinna* showed significant antimicrobial activity against *Z. mobilis*, *E. coli*, and *S. aureus*, with minimum inhibition zones (MIZ) of 24, 22, and 20 mm, respectively. *C. limon* strongly inhibited all the tested pathogenic bacteria with maximum and minimum MIZ of 32 and 17 mm. *A. concinna* silver nanoparticles also exhibited potent antimicrobial activity. Both extracts showed substantial antioxidant, antihyperlipidemic, antidiabetic, anticancer (MCF-7), and anti-urease (antiulcer) properties. To conclude, these plants can be used to treat hyperlipidemia, diabetes, cancer, and gastrointestinal ulcers. They can also serve as antimicrobial and antioxidant agents. Thus, the studied plants must be exploited cost-effectively to generate therapeutic drugs for various diseases.

## 1. Introduction

Medicinal plants are utilized as disease remedials, leading to minor side effects [1]. They are currently used to manage diseases of human beings. They are used by the worldwide population as primary health care [2]. They are thus rich in phyto-substances with positive pharmacological aspects. Some of them are alkaloids, glycosides, flavonoids, phenols, insecticides, polyphenols, vitamins, steroids, terpenoids, saponins, tannins, and coumarins [1,3,4]. For the synthesis of polysaccharides and glycosides, the pentose pathway is followed. However, the shikimate pathway is utilized for aromatic alkaloids, phenols, and tannins. The pathway followed for alkaloids and phenols is acetate–malonate, while the mevalonate route is considered for steroids and terpenes [2,5]. Phytocompounds of medicinal plants can serve as curing agents of various diseases such as diabetes, cancer, blood pressure, etc. [2,6,7].

As per the ethnobotanical literature of phytotherapy of Indian medicinal plants, species such as *A. concinna* and *C. limon* are used by the communities for disease treatment as well as in modern medicine [8]. In Ayurveda, the fruits of *A. concinna* are used for promoting hair growth [9]. Citrus peels reduce coughs, diabetes, phlegm, and are thus used as antithyroidal, antiperoxidative, and hypoglycemic agents. This could be ascribed to the presence of total polyphenols in considerable amounts [10]. The medicinal plants have been extensively used in drug and pharmaceutical industries [4]. A whole medicinal plant or a part of it, such as leaves, seeds, roots, flowers, stems, bark, etc., may be utilized, but the phytochemical contents vary greatly [11]. Cardiovascular diseases and obesity are currently increasing owing to the food intake rich in fat and cholesterol. The pancreatic lipase is inactive in both cases. Thus, the search for phytocompounds that can overcome this issue is necessary [12]. The occurrence of gastrointestinal and urinary tract infections is also increasing due to dysfunction of the urease enzyme, resulting in an increase of pH owing to ammonia production and better growth conditions for *Helicobacter pylori*. Thus, the inhibition of urease by phyto-substances could be a better option [13].

Infectious diseases are causing morbidity in various countries, especially in underdeveloped countries owing to poverty, augmentation of multiple drug resistance incidence, and increase of undesirable antibiotic side effects [14]. Thus, medicinal plants can be exploited to solve these problems as excellent antimicrobial agents because they possess various phytochemicals [5]. For instance, citrus peels possess essential oils that can prevent the growth of pathogenic bacteria [10]. The phytochemicals from *A. concinna* and *Piper betel* leaves are bactericidal against pathogenic bacteria such as *E. coli*, *S. pyogenes*, *P. vulgaris*, and *S. aureus* [15]. It is thus vital to search for active phytosubstances able to cure pathogenic bacterial infections, since most of these microorganisms are gaining resistance towards available antibiotics [16]. In the present study, *A. concinna* and *C. limon* were selected. The study focused on phytochemical screening, characterization, and biological activity evaluation of antibacterial, antidiabetic, antioxidant, hypocholesterolemic, and anticancer activities. In addition, the use of *A. concinna* leaf extract as a reducing agent for silver nanoparticles’ preparation was also highlighted.

## 2. Materials and Methods

### 2.1. Plants, Microorganisms, Cell Lines, and Chemicals

*A. concinna* and *C. limon* used in the present investigation were collected from the University of Agriculture Science (Dharwad, India), and brought to the biochemistry laboratory. The bacterial strains used were *B. cereus*, *E. coli*, *Z. mobilis*, *B. subtilis*, *M. luteus*, *P. aeruginosa*, and *S. aureus.* They were obtained from the Microbial Type Culture Collection (Chandigarh, India). The MCF-7 cell line was bought from the National Centre for Cell Sciences (Pune, India). The chemicals such as DMSO, DPPH, MTT, BSA, p-nitrophenyl palmitate, etc., were bought from Himedia (New Delhi, India) and Sigma-Aldrich Pvt Ltd. (St. Louis, MO, USA), Bangalore, Karnataka. All the reagents utilized in the present study were of analytical grade.

### 2.2. Extraction and Quantification of the Phytochemicals

The plants were cleaned with tap water, rinsed with distilled water, dried under an aseptic condition for a week at 30 ± 3 °C, and powdered with an electrical stainless steel grinder. Ten grams of the powdered plant sample of *A. concinna* was taken and it was extracted with 150 mL methanol (95%, *v*/*v*) using a Soxhlet apparatus at 60 °C for 6 h. The extract obtained was concentrated using a rotary evaporator so that the solvent evaporates, leaving behind the pure sample. After evaporation, the crude extract stock solution with the concentration of 94.11 mg/mL was stored in amber-colored glass bottle in the refrigerator at 4 °C. The powdered peel of *C. limon* was treated as above. The profiling of phytochemicals was conducted to confirm the presence of the bioactive secondary metabolites present in the extracts. Various phytochemicals (alkaloids, flavonoids, glycosides, coumarins, diterpenes, steroids, saponins, phlobatannins, tannins, phenols, triterpenoids, catechins, and anthocyanosides) were analyzed for their presence. Quantitative analysis was also carried out for alkaloids, flavonoids, and phenols [17,18]. The plant extracts were kept at 4 °C for future investigations.

### 2.3. Identification of Functionally Organic Groups in the Phytochemicals

The chemical bonds and organic functional groups were given by FTIR spectroscopy [19] and high performance liquid chromatography (HPLC) according to Sundararajan et al.’s procedure [20].

### 2.4. Antibacterial Action of A. concinna and C. limon Extracts

#### 2.4.1. Antibacterial Activity Studies and Minimum Inhibitory Concentration (MIC) Investigations

The inoculums were prepared from *B. cereus*, *B. subtilis*, *M. luteus*, *P. aeruginosa*, *Z. mobilis*, *E. coli*, and *S. aureus* using nutrient broth. The incubation was carried out overnight at 37 °C. For the agar well assay, microbial culture (0.1 mL) was spread on each Muller Hinton agar plate, and then the 6 mm wells were made in agar plates with a borer. Then, 100 µL of *A. concinna* or *C. limon* extract was added in each hole and left undisturbed for 60 min for proper extract diffusion. Clear zones were noted in mm after overnight incubation at 37 °C. Gentamicin, an unfilled well, and dimethyl sulphoxide were utilized as positive, neutral, and negative controls, respectively [15,18].

MIC was investigated using the concentration range of 50, 100, and 150 µg/mL. Dimethyl sulphoxide (DMSO) was used as a solvent to dilute plant extracts. Then, 1 mL of *A. concinna* and *C. limon* extract was mixed with 5 mL nutrient broth separately, and then it was inoculated with the pathogen, and then incubated at 37 °C. The turbidity was measured at 600 nm after a 24 h incubation period. Later, it was streaked on a nutrient agar plate for examination of growth. When there was no visible growth on the nutrient agar plate, it was considered as the MIC of plant extract on that microorganism [21].

#### 2.4.2. Partial Purification of Bioactive Compounds and Antimicrobial Activity for Protein Fractions

The partial purification of proteins from the plant extracts were carried out with ammonium sulfate followed by dialysis [22]. The protein level was quantified by Lowry et al.’s procedure using BSA as standard [23]. The antimicrobial activity of protein fractions from plant extracts were studied as described earlier by the agar well assay with *B. cereus*, *M. luteus*, *Z. mobilis*, and *B. subtilis.*

#### 2.4.3. Silver Nanoparticles’ Production by *A. concinna* Extract

In this study, 100 mL of silver nitrate solutions of 1, 5, and 10 mM were prepared. The *A. concinna* extract samples of 0.1, 0.5, and 1 mg/mL were added. A positive reaction was indicated by a change from colorless to a brownish color. The spectrum scanning was then carried out between 200 and 800 nm. The synthesized nanoparticles were checked if they could inhibit *Z. mobilis* as described above.

### 2.5. Anticancer Activity of A. concinna Extract and C. limon Extracts

The cytotoxic activity of *A. concinna and C. limon* extracts on the MCF-7 cell line was assessed. The principle of MTT assay was followed [22].

### 2.6. Antioxidant Activity of A. concinna and C. limon Extracts

The present activity was studied using DPPH as highlighted by Sundararajan et al. [20].

### 2.7. Enzyme Inhibition Investigations

#### 2.7.1. α-Amylase and α-Glucosidase Inhibitions by *A. concinna* and *C. limon* Extracts

α-amylase and α-glucosidase inhibitory activities of *A. concinna* and *C. limon* extracts were studied. In this assay, if the plant extract has the ability to inhibit the enzyme, the enzyme cannot utilize the substrate and hence there will be minimum product formation. % inhibition of α-glucosidase/amylase = (Absorbance control − Absorbance sample)/Absorbance control × 100 [24].

#### 2.7.2. In Vitro Lipase Activity Inhibition by *A. concinna* and *C. limon* Extracts

The lipase activity of *A. concinna* and *C. limon* extracts was studied using in vitro lipase enzyme inhibitory activity method.

% inhibition of lipase = (Absorbance control − Absorbance sample)/Absorbance control × 100 [18].

#### 2.7.3. In Vitro Urease Activity Inhibition by *A. concinna* and *C. limon* Extracts

The urease activity of *A. concinna* and *C. limon* extracts was studied using in vitro urease inhibitory activity [25].

### 2.8. Inhibition Kinetics Studies of Amylase, Glycosidase, and Urease

Enzyme inhibition kinetics was evaluated by analyzing the various enzyme activity with *A. concinna* extract at different concentration ranges of the enzyme substrate. The α-amylase activity was carried out with 2–20 mg/mL starch. For glucosidase inhibition kinetics, different concentration ranges of pNPG (1–10 mM) were considered. Urea in the range of 10 to 100 mM was used for urease inhibition studies. The activity was quantified as above [18]. Km and Vmax were quantified by double reciprocal plot [26].

### 2.9. Statistical Analysis of Data

All the procedures were repeated 3 times. The experimental results are shown as mean ± standard deviation (SD). They were uploaded in SPSS and analyzed. *p* < 0.05 was statistically significant after ANOVA and DMRT.

## 3. Results and Discussion

### 3.1. Screening of Phytochemicals

Plants have more importance due to the presence of active ingredients or essential organic compounds [11]. In the present investigation, *A. concinna* and *C. limon* plants were analyzed. With methanol as the extraction solvent, the phyto-substances observed in both plants were alkaloids, flavonoids, saponins, steroids and triterpenoids, tannins, phenols, glycosides, quinones, coumarins, phlobatannins, and anthocyanosides. However, diterpenes were only present in *C. limon*, while catechins were absent in both medicinal plants (Table 1). Similarly, the methanol was the solvent of choice to extract polyphenol/flavonoids compared to nonpolar solvents such as hexane and acetone [27]. Thus, *A. concinna* and *C. limon* are capable of producing phyto-substances with medical properties and have the ability to synthesize aromatic substances such as phenolics (e.g., phenolic acids, flavonoids, quinones, coumarins, lignans, stilbenes, tannins), nitrogen compounds (alkaloids, amines), vitamins, terpenoids (including carotenoids), and some other endogenous metabolites [2,4]. Quantitative phytochemical analysis was carried and total phenol, alkaloid, and flavonoid contents in the plant extracts are summarized in Table 2. These phytocompounds were present in a significant amount in both plants, except alkaloids, which were found in trace quantity in *C. limon*.

### 3.2. Identification of Functionally Organic Groups in the Phytochemicals

#### 3.2.1. FTIRS Analysis of *A. concinna* and *C. limon* Extracts

**Figure 1:**
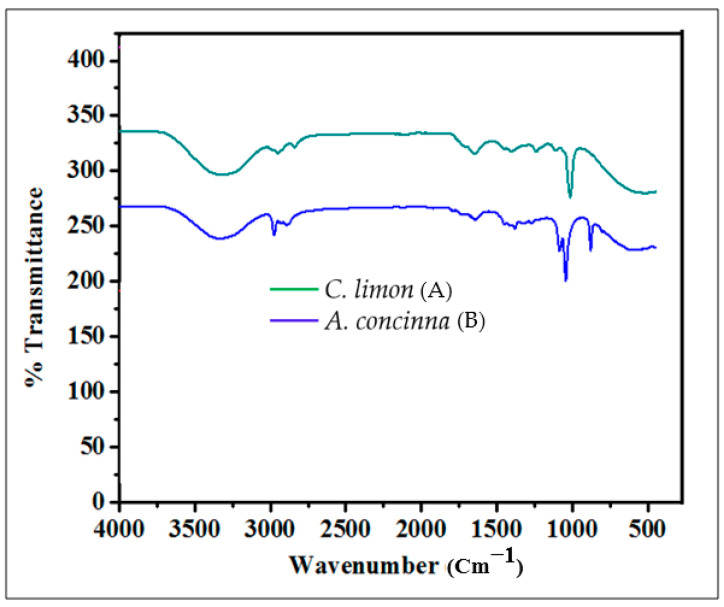
FTIR analysis of phytochemicals in the plant extracts. (**A**) FTIR trace of *Citrus limon*; (**B**) FTIR trace of *Acacia concinna.*

FTIRS was carried out on *A. concinna* and *C. limon* extracts. The peaks were as follows: *A. concinna* (Figure 1A). A broad peak at 723.79, 1000.00, 1100.00, 1230.47, 1383.74, 1461.23, 1620.91, 1700, 2900, 2960.92, 3300 cm^−1^ corresponds to the alkynes, C–O primary alcoholic group, C–N of amine, C–O alkyl aryl ether and aromatic ester, methyl symmetrical bending, methylene scissoring, C=C of aromatic compounds, ester group, C–H bond in methylene and methyl groups, aldehydic C–H group, and O–H of alcohols and phenols, respectively. The peaks for *C. limon* are depicted in Figure 1B, and a broad peak for alkynes, C–H bending vibration of substituted compounds, C–O primary alcoholic group, C=O in ester and lactone groups, aldehydic C–H, O–H in alcohols and phenols, C–H of alkanes, was observed at 723.79, 876.86, 1000.00, 1739.90, 2960.92, 3000, and 3010.96 cm^−1^, respectively. Bagewadi et al. helped to interpret the above results [18]. Therefore, based on the FTIR spectrum, various organic compounds (viz. aromatic compounds, alcohols, lactone, phenols, esters, alkanes, amines, alkynes, terpene, etc.) were observed in the plant extracts and corresponded to the phytochemicals seen.

#### 3.2.2. Determination of Quercetin from *A. concinna* Extract

**Figure 2:**
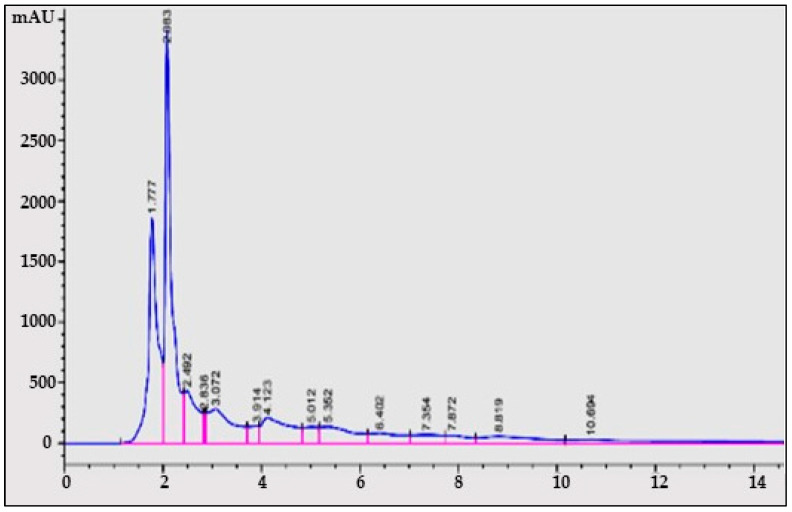
HPLC chromatograph of quercetin from *A. concinna* extract. The pink coloured lines represent the retention/peak values.

For determination of quercetin, methanol:water (38:62 *v*/*v*) was used as the mobile phase with 1 mL/min elution flow rate, and UV wavelength (λ) = 220 nm. A C-18 column was used for the quantification and identification of quercetin acid. With gallic acid as standard, the retention time for quercetin was 1.8 min as shown by the HPLC chromatograms from *A. concinna* extract (Figure 2).

#### 3.2.3. Determination of Compounds from Plant *C. limon* Extract

**Figure 3:**
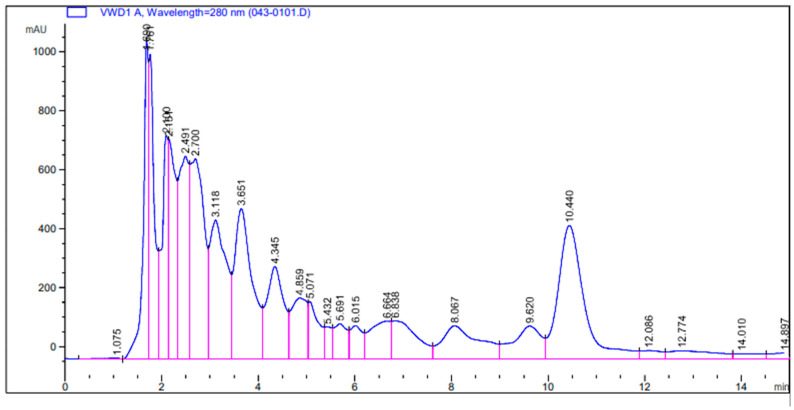
HPLC chromatograph of vanillin, quercetin, gallic acid, and caffeine from *C. limon* extract. The pink coloured lines represent the retention/peak values.

For determination of vanillin, gallic acid, caffeine, and quercetin, methanol:water (10:90, 25:25, 60:40, and 38:62 *v*/*v*) was used as the mobile phase with 1, 0.7, 1, and 1 mL/min as elution flow rates, and λ of 220, 270, 272, and 280 nm UV wavelength, respectively. C-18 was used for the quantification and identification of compounds. The compounds determined from *C. limon* were vanillin, quercetin, gallic acid, and caffeine, with retention time of 2.49, 1.8, 2.3, and 4.8 min, respectively (Figure 3).

### 3.3. Antibacterial Activity of A. concinna and C. limon Extracts and Determination of Minimum Inhibitory Concentration

Microbial species produce enterotoxins and exotoxins responsible for human diseases. Medicinal plants were reported to possess various compounds able to cure various diseases caused by microorganisms [15]. In the present study, antibacterial activity of methanolic extracts was performed by an agar well diffusion procedure on three Gram-negative (*E. coli*, *Z. mobilis*, *P. aeruginosa*) and four Gram-positive bacteria (*B. subtilis*, *B. cereus*, *S. aureus*, and *M. luteus*). Table 3 shows the zone of clearance of various extracts. The phytochemicals such as steroids obtained in a huge amount were responsible for antibacterial activity observed. For instance, the sterol acts by pore formation in the bacterial cell wall leading to its death [28]. The antibacterial action may also be due to the occurrence of flavonoids and polyphenols in the extracts present in high concentrations. Similar results have been reported by Chakraborty and Shah [15]. David and Sudarsanam [14] reported that bacteria might be killed by tannins after forming complex soluble substances with bacterial membrane proteins.

*C. limon*, commonly known as lemon, is bactericidal owing to alkaloids present in a significant amount in different parts of lemon [29]. In the same vein, the lack of antimicrobial effect by plant extracts may be ascribed to the solvent used, as some prefer polar and others non-polar solvents. The plant age, astringent aspects, harvesting time, and extraction procedure may also be responsible for inactive effects of the same phytocompounds [5,30]. These extracts can be used to cure diseases such as pneumonia, skin infections, and meningitis caused by *S. aureus*, and gastroenteritis, neonatal meningitis, and urinary tract infections caused by *E. coli* [28]. The bacterial growth prevention by the phytochemicals can be ascribed to inhibition of enzymes and nucleic acid biosynthesis by them [31].

Minimum inhibitory concentration (MIC) investigation of the *A. concinna* and *C. limon* extracts was quantified against investigated pathogenic bacteria. The MIC values observed against the assessed pathogenic microorganisms were 50, 100, or 150 µg/mL (Table 4). Similarly, MIC of 50 µg/mL against *C. krusei* and *E. coli*, 75 µg/mL against *S. aureus, K. pneumoniae*, and *B. cereus*, and 100 µg/mL against *C. tropicalis*, *C. kefyr*, and *C. albicans* were noticed [14].

### 3.4. Partial Purification of Bioactive Compounds and Antimicrobial Activity for Protein Fractions

Partial purification with 40% ammonium sulfate saturation followed by dialysis was used. The total protein in 2 mL observed after the dialysis step was 0.60, and 1.31 mg for *C. limon* and *A. concinna*, respectively.

### 3.5. Synthesis of Silver Nanoparticles and Antimicrobial Activity of A. concinna

**Figure 4:**
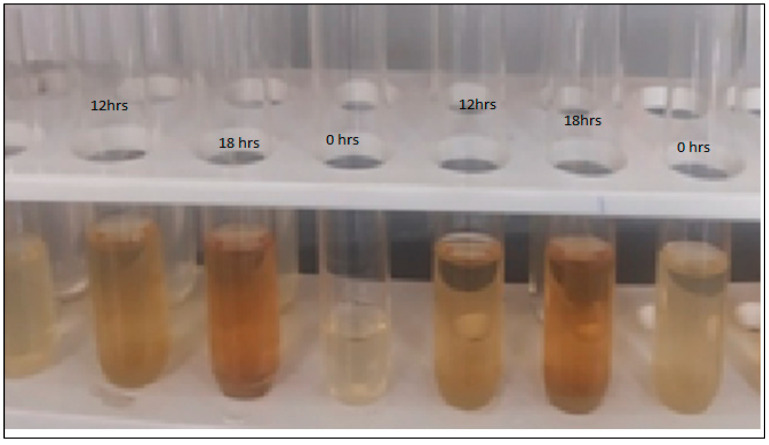
Incubation of silver nitrate solution with *A. concinna* leaf extract.

**Figure 5:**
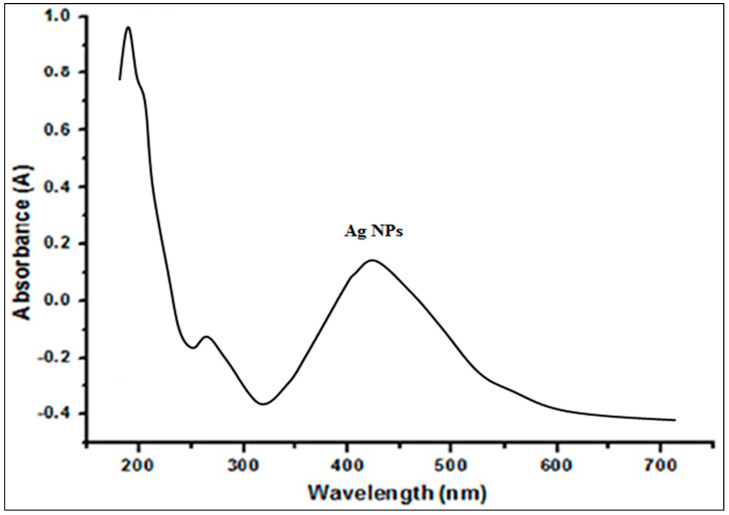
UV–vis spectrum showing formation of silver nanoparticles.

**Figure 6:**
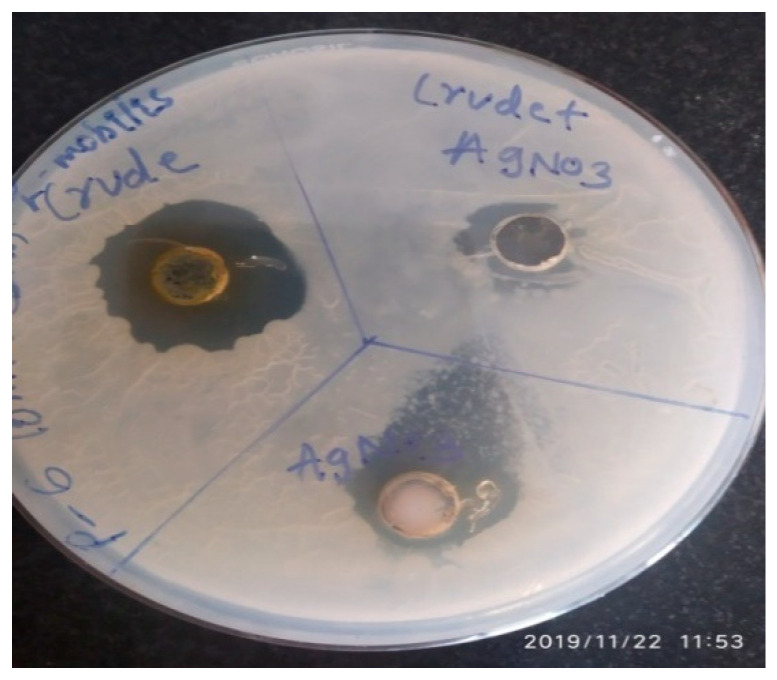
Antimicrobial activity of synthesized nanoparticles by *A. concinna* extract against *Z. mobilis*.

Silver nanoparticles can act as an antibacterial agents [32,33]. Leaf extracts of *A. concinna* were utilized as reducing agents for silver nanoparticles’ production. After 18 h of incubation, AgNO_3_ solution, which was incubated with *A. concinna* extract, changed to brown (Figure 4). The characteristic absorbance was measured at 200–400 nm in the UV–vis spectrum for the confirmation of silver nanoparticles (Figure 5). Silver nanoparticles synthesized using *A. concinna* extract exhibited antimicrobial activities against *Z. mobilis*, which is a pathogenic microorganism (Figure 6). Similarly, the Ag-ZnO nanocomposites produced by *Verbascum speciosum* were able to kill the most infectious pathogenic *S. aureus* and *E. coli* [34]. Likewise, a nanocomposite composed of Ag_2_O_3_ and ZnO was able to inhibit both Gram-positive and -negative pathogenic bacteria [35]. The bacterial cells are killed by direct interaction between nanoparticles and bacterial cells, leading to disorganization of bacteria caused by positive ion release or by interaction between positive ions and negative bacterial cell wall, resulting in death of microbial cells due to electrostatic interactions [36,37].

### 3.6. Anticancer Activity of A. concinna and C. limon Extracts

Various phytochemicals were reported to manage and treat effectively cancer diseases [29,38]. The cytotoxicity of the *A. concinna* extract was assessed on the MCF-7 cell line. At 100–500 µg/mL, the % of cell viability was noticed to decrease with the increase in the concentration of *A. concinna* extracts and *C. limon* extracts (Table 5 and Table 6). The IC_50_ value calculated for *A. concinna* extract was 171.25 μg/mL and *C. limon* extracts was 98.61 μg/mL. The plant extract may thus exhibit anticancer activity, and this could be ascribed to the alkaloids present [1,38,39]. Diab [29] reported the strongest antioxidant against HL-60 cells by the lemon peel extract as highlighted by the highest DPPH radical scavenging influence, with EC50 of 42.97 μg extract /mL. The extracts of *Pogostemon heyneanus* and *Plectranthus amboinicus* were effective against MCF-7 cells [40]. The leaf extract of *D. inoxia* showed anticancer properties owing to alkaloids, phenolic compounds, and steroids’ presence [18]. Similar observation of possessing cytotoxic properties by phyto-substances due to steroids and alkaloids were reported with similar cell lines to this study [41]. The phytocompounds may act synergistically with anticancer agents to conquer the resistance of these agents. The utilization of phyto-substances may also lead to the use of a low dosage of anticancer drugs [18].

### 3.7. Antioxidant Effect of A. concinna and C. limon Extracts

Antioxidants from a plant origin may prevent various severe consequences to humans, such as prevention of cancer and heart diseases [1]. The antioxidant activity of *A. concinna* and *C. limon* extracts was assessed with the DPPH procedure. The percentage scavenging activities calculated were 99.9 and 75.1% for *A. concinna* and *C. limon*, respectively. This important scavenging action could be ascribed to the phyto-substances present, especially those with OH groups [25,27]. Similarly, the presence of flavonoids and phenolic compounds in *D. inoxia* were responsible for the antioxidant capacity observed in *D. inoxia* [27,42]. Hegazy and Ibrahim [27] reported an excellent antioxidant effect of the juice from orange peels. The antioxidant effect was also shown by the extracts of grapefruit and lemon peels during in vitro investigations [29]. Thus, plant antioxidants may prevent various diseases (like cancer disorders) by preventing the free radicals/cell damage action.

### 3.8. Enzyme Inhibition Activities

#### 3.8.1. α-Amylase and α-Glucosidase Inhibition by *A. concinna* and *C. limon* Extracts

Diabetes can be controlled by inhibiting the important enzymes involved in carbohydrate metabolism, such as α-glucosidase and α-amylase [1,39]. In vitro antidiabetic action of *A. concinna* and *C. limon* extracts was carried out using alpha amylase and glucosidase enzymes. The inhibition of amylotic activity was observed with *A. concinna* (100.0%) and *C. limon* (73.2%). The glucosidase activity was also optimally inhibited by the extract of *A. concinna* (100%) and *C. limon* (95.8%). The same enzymes were also inhibited by the extracts obtained from *D. inoxia* [18] and *Sapium ellipticum* [24]. This inhibition can be ascribed to the occurrence of OH groups in phenols and flavonoids that hydroxylate and substitute the carbohydrate β-bonds [7]. In addition, the *C. limon* peel extract with hexane was reported to possess an important activity compared to the commercial Glimepiride, which is used as standard antidiabetic agent [10]. Thus, *A. concinna* and *C. limon* plants can be cost-effectively exploited as curative agents of diabetes.

#### 3.8.2. In Vitro Lipase Activity Inhibition by *A. concinna* and *C. limon* Extracts

Obesity may arise owing to the dietary fat absorption in a significant amount, leading to the inability of the lipase of the pancreas to digest fat [12]. In vitro fat metabolism activity of *A. concinna* and *C. limon* extracts was carried out by analyzing the lipase inhibitory effect. Maximum lipase inhibition of 100% was noticed for the extracts from both plants. The lipid hydrolysis was also inhibited by the extracts from *D. inoxia* [18]. The juice obtained from the fruit of *Cudrania tricuspidata* strongly inhibited the pancreatic lipase [12]. The inhibition of lipase can be attributed to flavonoids such as polymethoxyflavones, as suggested by the Zeng et al. [3]. The extracts of *A. concinna* and *C. limon* can therefore be useful for the management of obesity.

#### 3.8.3. In Vitro Urease Activity Inhibition by *A. concinna* and *C. limon* Extracts

The urease action leads to ammonia secretion (increase of pH), thereby favoring the growth of the pathogenic bacterium *Helicobacter pylori*. This present pathogen is responsible for various diseases such as gastrointestinal and urinary tract infections [13]. The urease activity was completely inhibited by the extract of *C. limon* and *A. concinna*. These findings are in contrast to previous studies, which reported weak inhibition of urease activity by *Geranium purpureum* [25] and *D. inoxia* [18] extracts. A vital anti-urease activity was reported by Shah [43] for the phytochemicals extracted from *Asparagus gracilis*. *A. concinna* and *C. limon* extracts can inhibit the urease activity, thereby curing various diseases, such as throat infections, dysuria, common cold, urinary troubles, asthma, ulcer, and cough. Thus, urease inhibition is also known as a better option to prevent *H. pylori* survival [13].

### 3.9. Inhibition Kinetics Studies of Amylase, Glycosidase, and Urease

**Figure 7:**
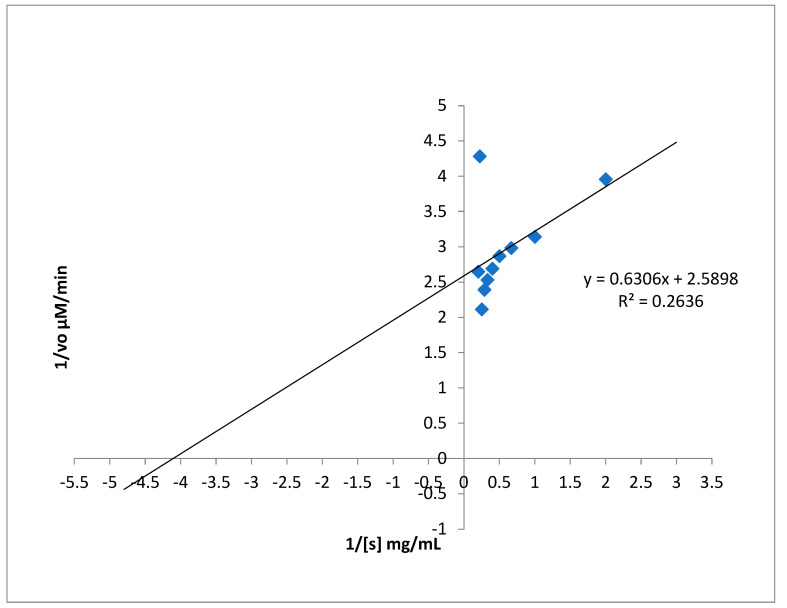
Inhibition kinetics of amylase activity.

**Figure 8:**
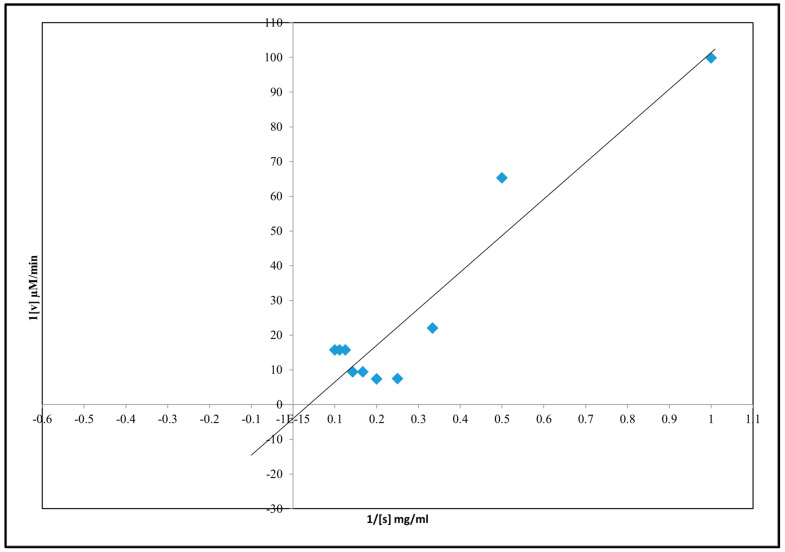
Inhibition kinetics of α-glucosidase activity.

**Figure 9:**
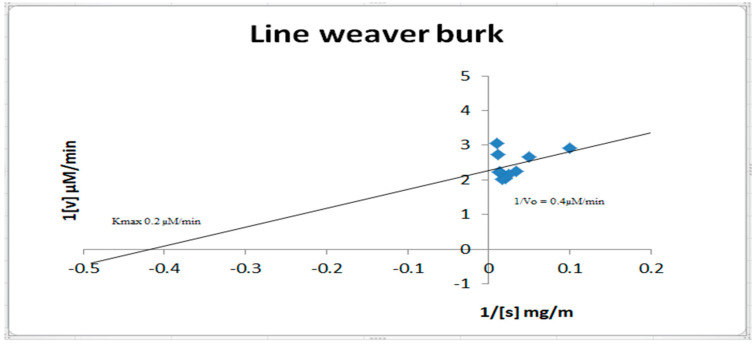
Inhibition kinetics of urease activity.

Kinetics inhibition of α-amylase, urease, and α-glucosidase by *A. concinna* extract were carried out by varying the different substrate concentrations. The substrates used for α-amylase, α-glucosidase, and urease activities were starch (2–20 mg/mL), pNPG (1–10 mM), and urea (1–100 mM), respectively. *K*m and *V*max were calculated. The results are in Figure 7, Figure 8 and Figure 9. For α-amylase, Vmax was 0.2 µM/min and Km 1 mg/mL (Figure 7). Vmax was 0.05 µM/min and Km was 0.9 mg for α-glucosidase (Figure 8). For urease, the extract was found with a Vmax of 0.2 µM/min and Km of 0.9 mg/mL (Figure 9).

## 4. Conclusions

In the present investigation, the extraction of the phytocompounds of *A. concinna* and *C. limon* was performed. The plant extracts showed significant antimicrobial activities against studied pathogenic bacteria owing to the presence of phytocompounds such as alkaloids, flavonoids, and phenols. The inhibition of α-amylase, α-glucosidase, and urease enzymes showed that the plants could be used in the management of diabetes and acid-peptic disorders. Silver nanoparticles synthesized using *A. concinna* extract exhibited antimicrobial activities against pathogenic *Z. mobilis*. The current study demonstrates that the bioactive chemicals found in *A. concinna* and *C. limon* can be used in clinical and pharmacological applications to treat a variety of ailments such as cancer, diabetes, gastrointestinal ulcers, and bacterial infections. However, further research is warranted to elucidate the exact mechanism of action of these medicinal plants.

## Figures and Tables

**Table 1:** Phytochemicals observed in *A. concinna* and *C. limon* after methanol extraction. +: present, −: absent.

Phytoconstituents	*A. concinna*	*C. limon*
Alkaloids	+	+
Flavonoids	+	+
Saponins	+	+
Steroids and triterpenoids	+	+
Tannins	+	+
Phenols	+	+
Glycosides	+	+
Quinones	+	+
Coumarins	+	+
Diterpenes	−	+
Phlobatannins	+	+
Anthocyanosides	+	+
Catechins	−	−

**Table 2:** Concentration of some phytochemicals observed from plant extracts.

Medicinal Plant	Alkaloids (mg/L)	Flavonoids (mg/L)	Phenols (mg/L)
*A. concinna*	0.1860 ± 0.0001	0.2768 ± 0.0003	0.2431 ± 0.0002
*C. limon*	0.0002 ± 0.0000	0.1752 ± 0.0001	0.3401 ± 0.0007

**Table 3:** Zone of bacterial clearance of various extracts in mm.

Medicinal Plant	*E. coli*	*B. subtilis*	*Z. mobilis*	*M. luteus*	*S. aureus*	*B. cereus*	*P. aeruginosa*
*A. concinna*	22 ± 0.763	7 ± 0.123	24 ± 0.801	7 ± 0.285	20 ± 0.365	5 ± 0.169	5 ± 0.401
*C. limon*	22 ± 0.671	17 ± 0.249	22 ± 0.593	32 ± 0.582	27 ± 0.801	22 ± 0.467	21 ± 0.397

**Table 4:** MIC of *A. concinna* and *C. limon* at 600 nm.

***A. concinna***	***E. coli***	***B. subtilis***	***Z. mobilis***	***M. luteus***	***S. aureus***	***P. aeruginosa***
50 µg/mL	−	−	−	−	−	−
100 µg/mL	−	−	−	−	−	−
150 µg/mL	−	−	−	−	−	−
***C. limon***						
50 µg/mL	+	−	−	+	−	−
100 µg/mL	−	−	−	−	−	−
150 µg/mL	−	−	−	−	−	−

**Table 5:** Anticancer activity of *A. concinna* extract.

	Blank	Untreated	Cisplatin 15 µg/mL	100	200	300	400	500 µg/mL
Reading 1	0.009	0.63	0.068	0.375	0.229	0.19	0.011	0.007
Reading 2	0.005	0.69	0.068	0.387	0.202	0.172	0.017	0.009
Mean OD	0.007	0.685	0.068	0.381	0.2155	0.181	0.014	0.008
Mean OD-Mean blank		0.678	0.0681	0.374	0.2085	0.174	0.007	0.001
Standard deviation		0.0169706	0	0.008485	0.019092	0.012728	0.004243	0.001414
Standard Error		0.012	0	0.006	0.0135	0.009	0.003	0.001
% Standard error		1.7699115	0	0.884956	1.99115	1.327434	0.442478	0.1474
% Viability		100	8.997050	55.16224	30.75221	25.66372	1.032448	0.147493

**Table 6:** Anticancer activity of *C. limon* extracts.

	Blank	Untreated	Cisplatin 15 µg/mL	100 µg/mL	200 µg/mL	300 µg/mL	400 µg/mL	500 µg/mL
Reading 1	0.009	0.63	0.068	0.365	0.21	0.194	0.02	0.008
Reading 2	0.005	0.69	0.068	0.367	0.204	0.182	0.022	0.007
Mean OD	0.007	0.66	0.068	0.366	0.207	0.188	0.021	0.007
Mean OD-Mean blank		0.653	0.061	0.359	0.2	0.181	0.014	0.0005
Standard deviation		0.04	0	0.001	0.004	0.008	0.001	0
Standard Error		0.03	0	0.001	0.003	0.006	0.001	0.0005
% Standard error		4.59	0	0.15	0.45	0.91	0.15	0.07
% Viability		100	9.34	54.97	30.62	27.71	2.14	0.076

## Data Availability

Not applicable.

## Sample Availability

Samples of the compounds are available from the authors.
